# Adoptive Transfer of Bone Marrow-Derived Monocytes Ameliorates *Schistosoma mansoni* -Induced Liver Fibrosis in Mice

**DOI:** 10.1038/s41598-019-42703-y

**Published:** 2019-04-23

**Authors:** Veruska Cintia Alexandrino de Souza, Danielle Maria Nascimento Moura, Maria Carolina Accioly Brelaz de Castro, Patrícia Torres Bozza, Ligia de Almeida Paiva, Camila Juliet Barbosa Fernandes, Renata Lins Carneiro Leão, Jéssica Paula Lucena, Roni Evencio de Araujo, Alex José de Melo Silva, Regina Celia Bressan Queiroz Figueiredo, Sheilla Andrade de Oliveira

**Affiliations:** 1 Instituto Aggeu Magalhães, Fundação Oswaldo Cruz, Recife, Pernambuco Brazil; 2 Centro Acadêmico de Vitória, Universidade Federal de Pernambuco, Vitória de Santo Antão, Pernambuco Brazil; 3 Laboratório de Imunofarmacologia, Instituto Oswaldo Cruz, Rio de Janeiro, Rio de Janeiro Brazil

**Keywords:** Cell delivery, Cell delivery, Immunotherapy, Immunotherapy

## Abstract

Liver diseases are a major health problem worldwide leading to high mortality rates and causing a considerable economic burden in many countries. Cellular therapies as potential treatments for liver diseases have proven beneficial in most of the conditions. In recent years, studies involving therapy with bone marrow cells have been implemented to promote liver regeneration and to reduce hepatic fibrosis, however identifying the cell population present in the bone marrow that is responsible for hepatic improvement after therapy is still necessary. The aim of the present study was the evaluation of the therapeutic efficacy of monocytes obtained from bone marrow in fibrosis resulting from *S*. *mansoni* infection in C57BL/6 mice. Monocytes were isolated by immunomagnetic separation and administered to the infected animals. The effects of treatment were evaluated through morphometric, biochemical, immunological and molecular analyzes. Monocyte therapy promoted reduction of liver fibrosis induced by *S*. *mansoni* infection, associated with a decrease in production of inflammatory and pro-fibrogenic mediators. In addition, monocyte infusion caused downregulation of factors associated with the M1 activation profile, as well as upregulation of M2reg markers. The findings altogether reinforce the hypothesis that the predominance of M2reg macrophages, producers of immunosuppressive cytokines, may favor the improvement of hepatic fibrosis in a preclinical model, through fibrous tissue remodeling, modulation of the inflammatory response and fibrogenesis.

## Introduction

Chronic liver diseases (CLD) represent a serious public health problem in the world. Continuous hepatic injury may result in alterations in liver repair processes, characterized by progressive formation of tissue fibrosis and changes in angiogenic architecture^1,2^. It is known that a variety of cell types and soluble mediators are involved in the course of CLD, integrating inflammatory processes, fibrosis and extracellular matrix components (ECM) remodeling^3^. Treatment of liver diseases depends on its specific cause, being associated, when possible, with the withdrawal of the aggressor stimulus^4^. Preclinical and clinical studies with different cell types have shown tissue and biochemical improvement following cellular therapy in liver lesions^5–7^. Good results have been achieved, but greater knowledge about the cellular population involved and their mechanism of action are needed.

*Schistosoma mansoni* infection continues to be one of the highly prevalent parasitic infections with important economic and public health consequences^8^. Chronic schistosomiasis is characterized by liver fibrosis resulting from host immune response to *S*. *mansoni* eggs, with formation of hepatic granuloma and systematized fibrous expansion of portal spaces (Symmer’s pipe-stem fibrosis)^8^. In this scenario, the therapeutic potential of different cell populations has been studied. Decrease of liver fibrosis was observed in mice infected by *S*. *mansoni* subjected to treatment with bone marrow mononuclear cells^9,10^ and mesenchymal stem cells^11^. In addition, an improvement in liver regeneration occurred when the animals were treated with cellular therapy associated with conventional chemotherapy^12,13^.

Monocyte/macrophage cells present in bone marrow may have an applicable therapeutic potential in liver disease due to their plasticity and known participation in several processes, in addition to inflammation and fibrogenesis in the resolution of fibrosis^14^. This functional plasticity of macrophages is driven by the immunological microenvironment, and can be activated by different pathways^15^. Intensive studies of liver injury in experimental models have sought to elucidate and illuminate ambiguities that describe the complex heterogeneity of monocyte and macrophages subsets in the liver^16,17^.

Macrophage populations may play a protective role^18^ through an active participation in hepatic repair by expressing metalloproteinases (MMPs)^19^ and inducing apoptosis of hepatic stellate cells (HSCs)^17^. In addition, previous results demonstrated that infusion of bone marrow monocytes in a murine model of CCl_4_-induced fibrosis promoted significant reduction in liver injury, associated with a decrease in pro-fibrogenic factors and oxidative stress^20^. Infusion of monocytes may be regulating important axes of the complex interaction between cells and the hepatic extracellular matrix, resulting in reduction of liver fibrosis. Here, we address the importance of cellular therapy with monocytes in an experimental model of liver fibrosis induced by *S*. *mansoni* infection.

## Results

### Therapy with monocytes reduces area and numerical density of granuloma and liver fibrosis induced by *S*. *mansoni* infection

**Figure 1:**
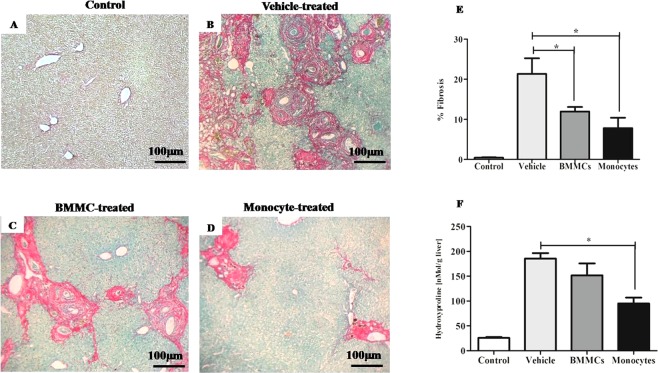
Reduction of liver fibrosis induced by *S*. *mansoni* infection after cell therapy. Representative images of fibrous tissue in liver sections sampled randomly and stained by Sirius red/fast green (100X magnification) in (**A**) healthy control mice, *S*. *mansoni*–infected mice 8 weeks after therapy with (**B**) vehicle, (**C**) Bone Marrow Mononuclear Cells (BMMCs) or (**D**) Monocytes. (**E**) The liver sections were examined by optical microscopy. Images were digitalized and analyzed by morphometry for quantification of fibrous tissue. (**F**) Fresh liver sample two months post-therapy was used for the determination of collagen, measured as hydroxyproline by spectrophotometry. Values are presented as means ± S.E. (n = 6) *P < 0.05. Statistical analysis was performed by Kruskal-Wallis, followed by Dunn *post-hoc* test.

**Figure 2:**
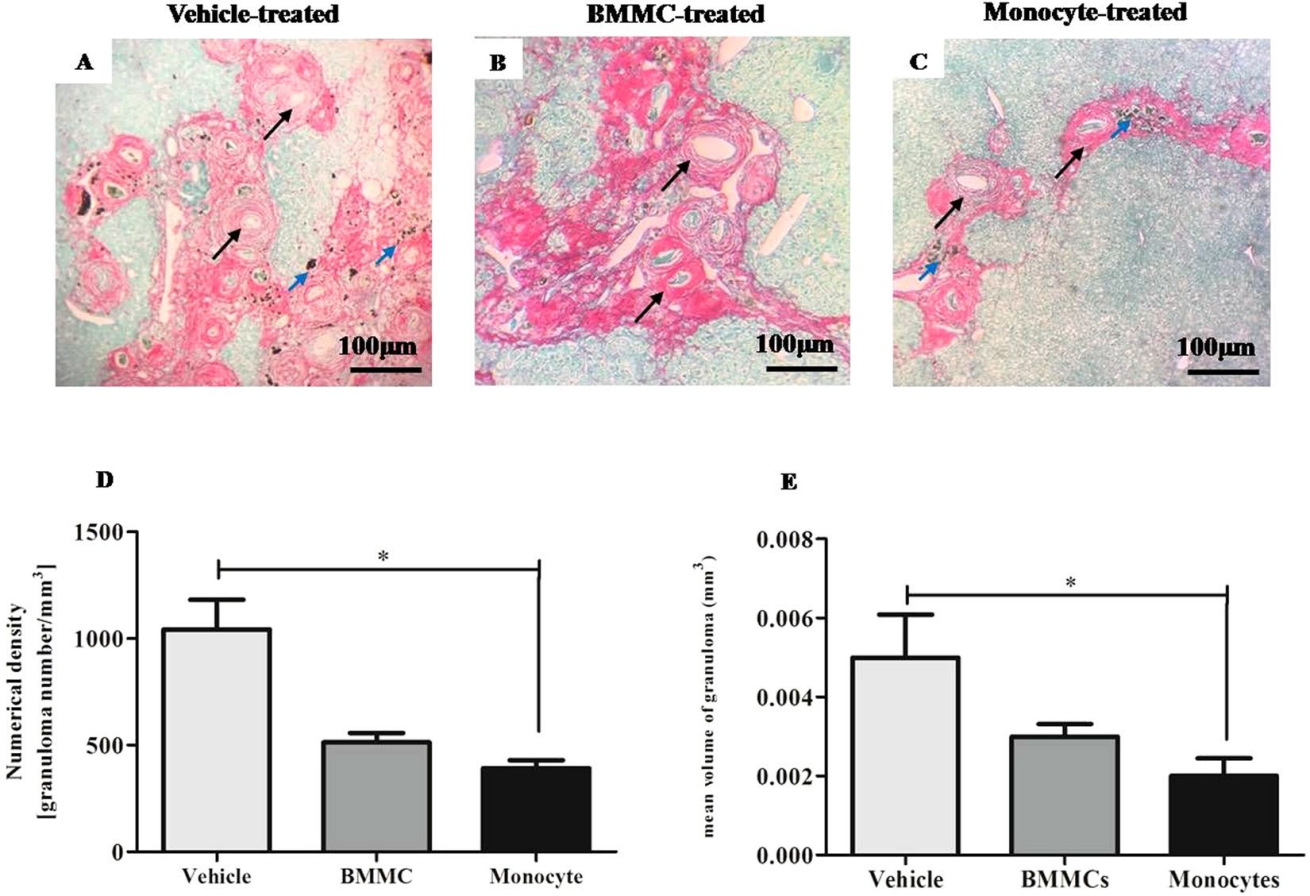
Reduction of granuloma number and volume after monocyte therapy. Liver sections sampled randomly and stained with Sirius red/fast green were examined by optical microscopy (100X magnification). Images of liver section of *S*. *mansoni*–infected mice 8 weeks after therapy with (**A**) vehicle, (**B**) Bone Marrow Mononuclear Cells (BMMCs) or (**C**) Monocytes, were digitalized and analyzed by morphometry for evaluation of (**D**) numerical density and (**E**) mean volume granulomas. Hepatic granulomas, well delimited and spherical shape which had the *S*. *mansoni* egg inside (black arrow) were considered during the measurement. Blue arrow – *Schistosoma* pigment. Values are presented as means ± S.E. (n = 6) *P < 0.05. Statistical analysis was performed by Kruskal-Wallis, followed by Dunn *post-hoc* test.

Morphometric analysis and hydroxyproline quantification showed that eight weeks after therapy, the monocyte- and Bone Marrow Mononuclear cells (BMMC)-treated groups had a statistically significant reduction of liver fibrosis (P < 0.05) (Fig. 1). The morphometric evaluation also showed a significant reduction of numerical density and granuloma volume eight weeks after monocyte transplantation (P < 0.05) (Fig. 2).

### Cellular therapy decreases production of inflammatory and pro-fibrogenic mediators

**Figure 3:**
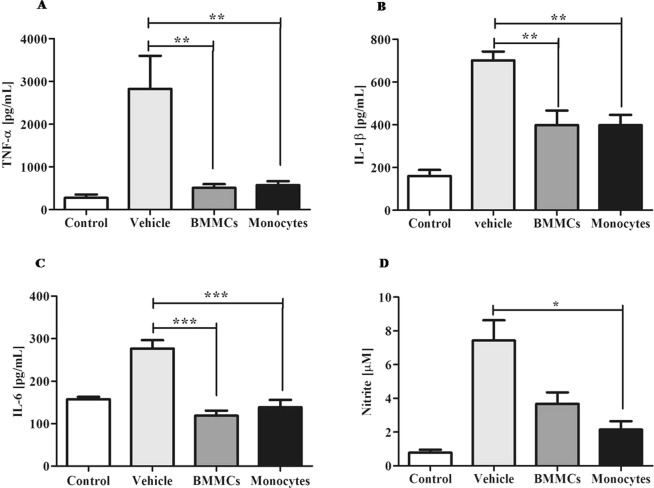
BMMCs and monocytes therapy decreases the levels of inflammatory cytokines. Cytokine levels were assessed in the soluble fraction of fragments of hepatic tissue of healthy control mice and *S*. *mansoni* –infected mice eight weeks after therapy with vehicle, bone marrow mononuclear cells (BMMCs) or monocytes. Profile of pro-inflammatory cytokines (**A**) TNF-α, (**B**) IL-1β and (**C**) IL-6 in a chronic model of schistosomiasis, measured by sandwich ELISA. Effects of cellular therapy in fragments of hepatic tissue after monocyte therapy in liver profile of (**D**) Nitrite. Values are presented as means ± S.E. (n = 6) *P < 0.05, **P < 0.01, ***P < 0.001. Statistical analysis was performed by Kruskal-Wallis, followed by Dunn *post-hoc* test.

Quantification of inflammatory mediators in liver fragments from a chronic model of schistosomiasis showed a significant decrease in TNF-α, IL-1β (P < 0.01) and IL-6 (P < 0.001) levels (Fig. 3A–C) after cell therapy. Our trials also showed that infusion of bone marrow-derived monocytes promoted a significant decrease in hepatic levels of nitrite after therapy when compared to animals treated with PZQ alone (P < 0.05) (Fig. 3D).

**Figure 4:**
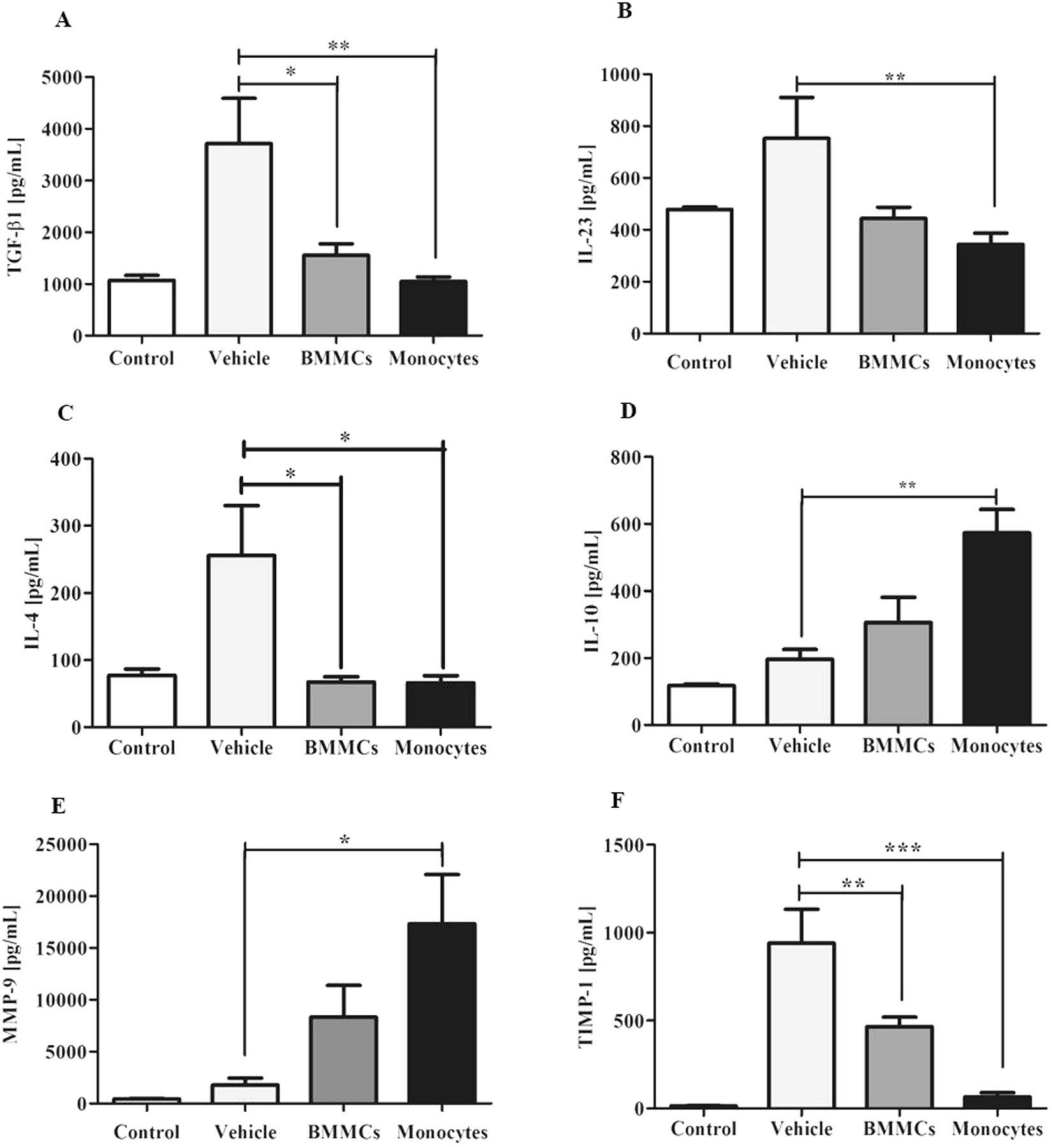
Cellular therapy alter regulatory mediators. Evaluation of effects of cell therapy in mediators (**A**) TGF-β1, (**B**) IL-23, (**C**) IL4, (**D**) IL-10, (**E**) MMP-9 and (**F**) TIMP-1 in fragments of hepatic tissue of Healthy control mice and *S*. *mansoni* –infected mice eight weeks after therapy with vehicle, bone marrow mononuclear cells (BMMCs) or monocytes. Measurement by sandwich ELISA. Values are presented as means ± S.E. (n = 6) *P < 0.05, **P < 0.01. Statistical analysis was performed by Kruskal-Wallis, followed by Dunn *post-hoc* test.

Significant reduction in levels of the pro-fibrogenic mediator TGF-β1 following monocyte (P < 0.01) and BMMCs (P < 0.05) therapy was also observed (Fig. 4A), while there were no changes in the hepatic levels of IL-13 and IL-17 (Supplementary Fig. S1). IL-23 (P < 0.01, Fig. 4B) and IL-4 (P < 0.05, Fig. 4C) cytokines presented significant reduction after monocyte infusion. Monocyte-treated animals also demonstrated significant increase in IL-10 hepatic levels compared to vehicle-treated animals eight weeks after therapy (Fig. 4D, P < 0.01).

### Increase in MMP-9 levels is accompanied by a decrease in TIMP-1 after bone marrow-derived monocyte transplantation in *S*. *mansoni* infected mice

The present study evidenced significant increase in MMP-9 levels after cell transplantation (P < 0.05) (Fig. 4E). Consistent with these results, significant reduction in hepatic TIMP-1 concentration was also observed both in monocyte (P < 0.001) and BMMCs (P < 0.01) therapy (Fig. 4F).

### α-SMA and galectin-3 expression levels are downregulated after monocyte therapy

**Figure 5:**
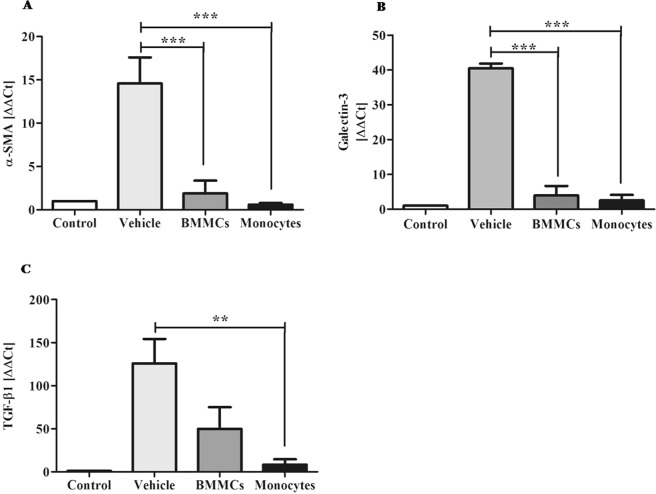
Decrease α-SMA and galectin-3 expression after cell therapy. Effects of cell therapy on liver expression levels of (**A**) α-SMA, (**B**) Galectin-3 and (**C**) TGF-β1, in chronic model of schistosomiasis, eight weeks after cell therapy. The targets were quantified by qPCR Values are presented as means ± S.E. (n = 6) *P < 0.05, ***P < 0.001. Statistical analysis was performed by Kruskal-Wallis, followed by Dunn *post-hoc* test.

Gene expression analysis by RT-qPCR showed significant reduction in expression of α-SMA, an important marker of activated HSCs, eight weeks after cell transplantation (P < 0.001) (Fig. 5A). Gal-3 and TGF-β1 expression was also significantly decreased in liver of animals submitted to cell transplantation with P < 0.001 and P < 0.01, respectively (Fig. 5B,C).

### Cellular therapy induces reduction in liver expression of markers associated with macrophages M1 activation profile

**Figure 6:**
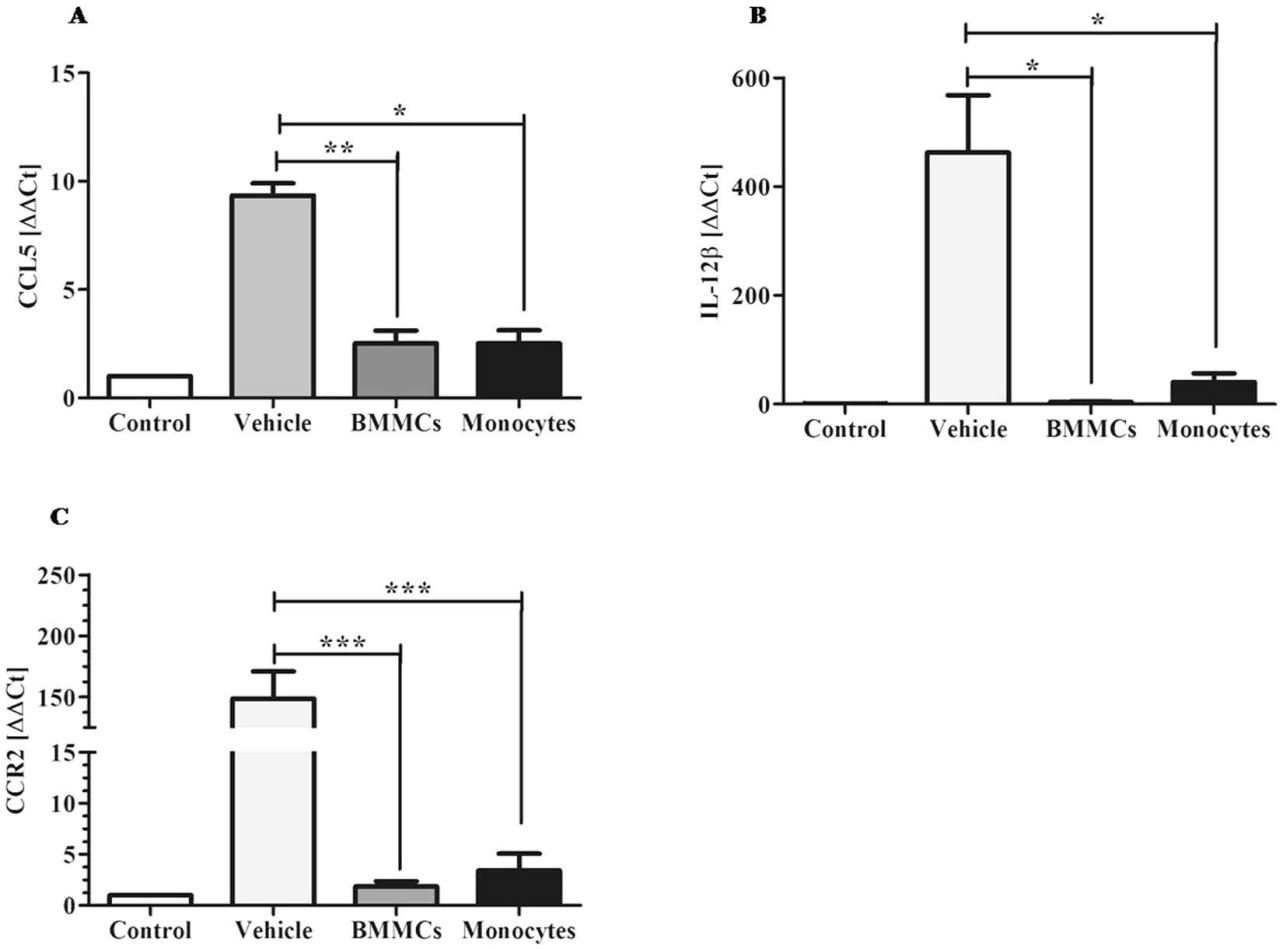
Effects of cell therapy on expression levels of M1 macrophages, in chronic schistosomiasis model. Liver expression of M1 profile markers (**A**) CCL5, (**B**) IL-12β and (**C**) CCR2 was quantified from liver tissue samples, by qPCR. Values are presented as means ± S.E. (n = 6) *P < 0.05, **P < 0.01, ***P < 0.001. Statistical analysis was performed by Kruskal-Wallis, followed by Dunn *post-hoc* test.

**Figure 7:**
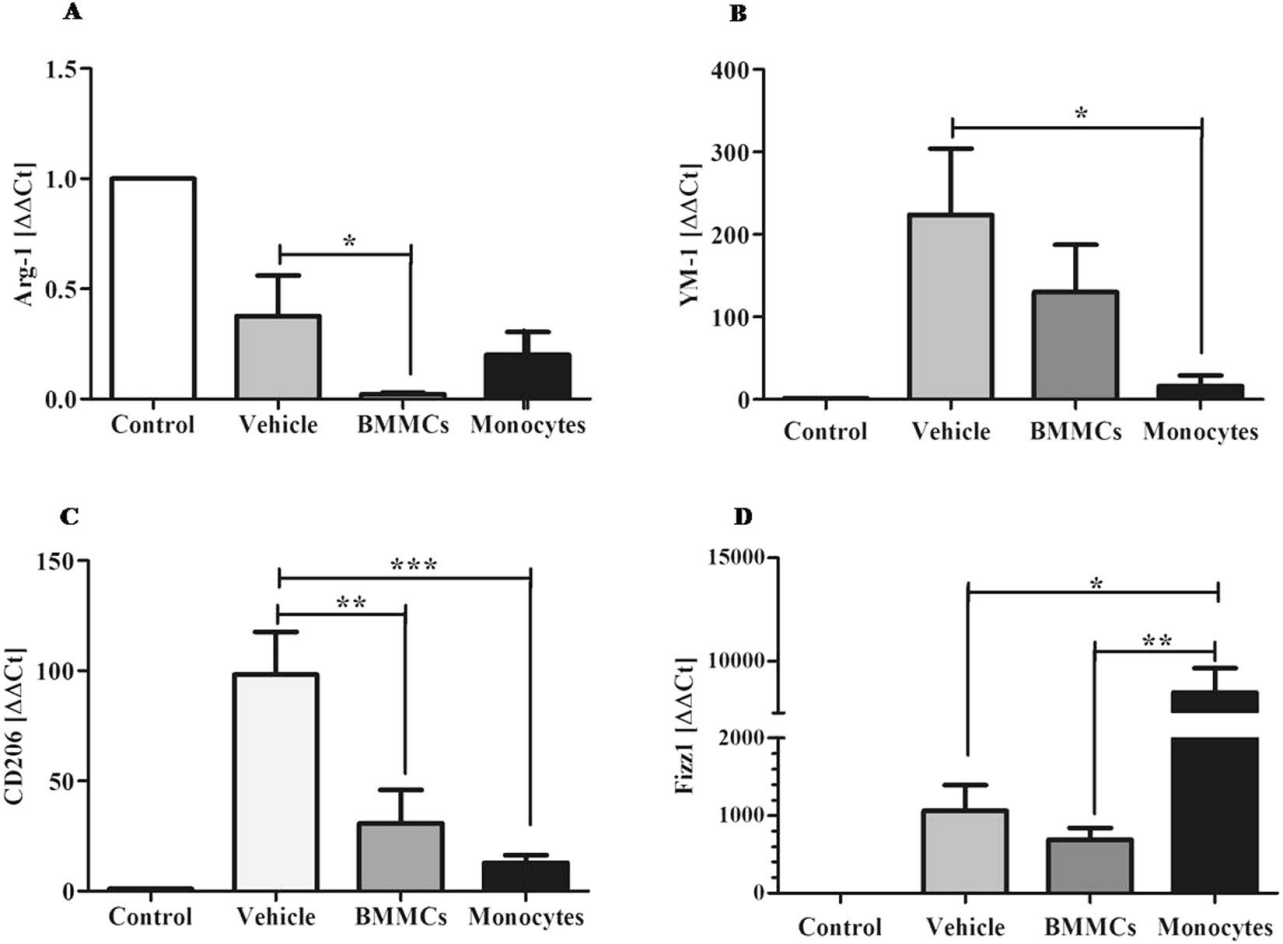
Effects of cell therapy on expression levels of M2 macrophages markers in chronic schistosomiasis model. Liver expression of M2 profile markers (**A**) Arginase-1, (**B**) YM-1, (**C**) CD206 and (**D**) Fizz1, was quantified by qPCR Values are presented as means ± S.E. (n = 6) *P < 0.05, **P < 0.01, ***P < 0.001. Statistical analysis was performed by Kruskal-Wallis, followed by Dunn *post-hoc* test.

Significant reduction in expression of the markers CCL5 (P < 0.01 for BMMCs and P < 0.05 for monocytes) (Fig. 6A), IL-12β (P < 0.05) (Fig. 6B) and CCR2 (P < 0.001) (Fig. 6C) was observed eight weeks after cell therapy. Molecular study also showed significant reduction in expression of markers of M2 profile: Arg-1 (P < 0.05/BMMCs) (Fig. 7A), YM-1 (Fig. 7B) (P < 0.05 for monocytes) and CD206 (P < 0.01/BMMCs and P < 0.001/monocytes) (Fig. 7C). However, the Fizz1 marker was shown to be significantly increased after cell transplantation (P < 0.01/BMMCs and P < 0.05/vehicle) (Fig. 7D).

## Discussion

In chronic liver diseases, the removal of the aggressive stimulus associated with anti-fibrotic therapy can promote promising results. Praziquantel (PZQ) is the most indicated therapeutic alternative for schistosomiasis control. The massive use of this drug for the treatment of schistosomiasis resulted in the emergence of strains with temporary resistance to the drug, making the development of new therapeutic options necessary. Previous research from our group using a CCl_4_–induced chronic liver injury model^20^ has indicated the monocyte therapy as a promising therapeutic modality for chronic liver diseases. In the present work, in an effort to reinforce the anti-fibrogenic potential of monocytes, we sought to indicate the predominant macrophages subtypes involved in the improvement of hepatic fibrosis in a murine model of chronic schistosomiasis by evaluation of specific markers related to macrophage activation profiles.

Monocyte/macrophage cell lineage has been an interesting object of investigation in recent years, taking into account their role in inflammation and tissue repair^21^. Macrophages have as their native characteristics their heterogeneity and plasticity, determined by microenvironmental stimuli^14^. Inflammatory macrophages, which promote inflammation and liver fibrosis, are activated by Toll-like receptors (TLR), with release of inflammatory (TNF, IL-1β) and pro-fibrogenic mediators (TGF-β and Gal-3) as well as chemokines (MCP-1, CCL5). On the other hand, restorative macrophages are involved in resolution of inflammation, with participation of anti-inflammatory cytokines and anti-fibrogenic molecules (IL-10, metalloproteinases)^22^. This study showed that CD11b CD14^+^ monocytes isolated from bone marrow in association with PZQ chemotherapy seems promising to improve the effects of liver fibrosis caused in mice by chronic *S*. *mansoni* infection. These findings corroborate results obtained previously^20^, where improvement in morphological, biochemical and immunological parameters after monocyte therapy was verified in a murine model of CCl_4_-induced liver fibrosis. As shown in previous studies, monocyte transplantation in a murine model of liver injury induces migration of circulating macrophages into lesion areas^23^, with the increase in the number of Kupffer cells in hepatic tissue^20,23^. From this information, we tried to understand if there is a predominance of some profile of macrophages associated to the improvement of liver fibrosis.

Morphometric study of fibrous tissue and the volume of liver granulomas showed that the cell therapy proposed here was capable of producing anti-fibrogenic effects in the murine model of chronic schistosomiasis. Reduction of liver fibrosis corroborates the results obtained by measurement of hepatic pro-fibrotic (TIMP-1, TGF-β1) and anti-fibrotic factors (MMP-9). In our investigation, it was possible to detect a reduction in α-SMA liver expression after monocyte transplantation, suggesting that macrophages may contribute to decrease fibrogenic activity attributed to activated HSCs. Previous work has associated BMCs transplantation with reduction of liver fibrosis and decrease in number of α-SMA-positive activated HSCs^24^. A previously published study conducted by us showed, in a toxic model of chronic liver injury, significant reduction of α-SMA positive cells marked by immunohistochemistry^20^. Thus, the present study reinforces the anti-fibrogenic effect of monocyte transplantation by evaluating the relative levels of gene expression of this HSCs marker.

Mediators released from immune cells can promote the activation of HSC in injured liver, which, in turn, can regulate immune response and promote chronic inflammation. The present study showed that monocyte therapy combined with PZQ chemotherapy promoted hepatic reduction of inflammatory and fibrogenic mediators involved in liver damage and establishment of liver fibrosis pathogenesis due to myofibroblast differentiation^25^. Additionally, our results showed an increase in IL-10 production after monocyte therapy. This anti-inflammatory cytokine plays a central role in regulating immune response and attenuating inflammation, thus preventing host lesions, and has been strongly associated with macrophages of M2reg profile^14,26^. In the liver, it has a protective function during development of chronic injury^27^. IL-10 also has been reported as a suppressor cytokine in the HSCs-associated fibrogenic response^28^, inhibiting collagen production and TGF-β secretion^27^. These results corroborate the description presented in Ju and Tacke^16^, which suggests the participation of restorative macrophages in modulating inflammation and fibrosis.

Other cytokines as IL-4, IL-17 and IL-23 were also evaluated after monocyte therapy. One previous study has associated high levels of IL-17 to the immunomodulation processes of liver granuloma^29^, as well as HSCs activation^30^. Our findings corroborate previous studies that identified IL-23 acts as an important pro-inflammatory cytokine inducing the Th17 fibrogenic response^25,31^, and reinforces its known participation in the response produced by inflammatory M1macrophages^32^. IL-4, also evaluated in the present study, is an immunological mediator, plays an important role in pathogenesis of schistosomiasis, with participation in fibrosis and granulomas formation^33^. In addition, alternative M2 macrophages are driven by IL-4, associated with an anti-inflammatory and pro-fibrogenic response^34^. IL-4 induces M2a macrophages to synthesize proline, a collagen-forming amino acid, and appears to be involved in promoting collagen deposition and hepatic fibrosis^35^. The therapeutic modality proposed in the present study seems to be capable of acting on important fibrogenic pathways, and may be very promising for chronic liver diseases.

Interestingly, an increase in the fibrolytic enzyme MMP-9 levels was observed in this study. This result indicates, once again, the anti-fibrogenic role that monocyte infusion can exert against liver fibrosis, as reported previously^20,36^. A study conducted by Ramachandran *et al*.^22^ identified that a population of Ly6C^low^ macrophages have an anti-fibrotic role in liver, since they secrete MMPs. The research proposes that increasing the production and the fibrolytic activity of MMPs should be considered for the development of anti-fibrogenic therapeutic approaches^37^.

Galectin-3 plays an important role in cell proliferation, adhesion, differentiation, angiogenesis and apoptosis, activation of pro-fibrotic M2 macrophages, and more recently has been associated with pathogenesis of hepatic fibrosis^38^. During helminth infection by *S*. *mansoni*, Gal-3 is directly involved in modulation of inflammatory response, being highly expressed by Kupffer cells around *Schistosoma* eggs^39^. Pre-clinical studies have reported that Gal-3 inhibitors promote liver fibrosis resolution in different experimental models^40,41^. Oliveira *et al*.^42^ demonstrated that BMCs therapy was able to promote improvement of liver fibrosis resolution associated with decreased liver expression of Gal-3 in a murine model of cirrhosis induced by CCl_4_. In accordance to these studies, our results showed that monocyte therapy promoted significant reduction in Gal-3 gene expression. All together, these findings emphasize the role of Gal-3 in fibrosis development and supports that monocytes, when used as cell therapy, act on important fibrogenic pathways.

Several investigations have aimed to identify and correlate distinct functional macrophage subgroups involved in tissue repair processes^17,28,43^. In the present study, molecular markers associated with M1 and M2 macrophages profiles were evaluated after monocyte therapy for chronic liver injury caused by *S*. *mansoni*. In our findings, we observed a significant reduction in NO levels, as well as a decrease in CCl5 and IL-12β expression, markers associated with the classic profile of macrophage activation (M1). These results corroborate the findings^44^ of analyzes of inflammatory mediators associated with the M1 profile observed after monocyte transplantation. Wynn and Ramalingam^44^ described that M1 macrophages are associated with the initiation of pro-fibrotic processes through the activation of myofibroblasts.

Analyzes of mRNA levels indicated that cell transplantation led to the reduction of Arg-1, YM-1 and CD206 markers, which are associated with an M2 profile, more specifically with the M2a or M2 fibrogenic^36,45^. Arginase-1 (Arg-1) is considered a prototype marker of M2 macrophages, and several studies have suggested that this enzyme is involved in fibrogenesis processes^46,47^. In schistosomiasis, a large number of Arg-1+ macrophages are located around liver granulomas^48^. Beljaars *et al*.^49^ associated the high expression of CD206 and YM-1 with the areas of hepatic fibrosis induced in mice by CCl_4_.

Interestingly, the evaluation of mRNA expression levels in this study showed a significant increase in Fizz1 after monocyte therapy. The role of Fizz1 in Th2 immune response as well as in fibrogenesis is not yet clear. There is no consensus regarding the relationship of this marker to an M2 macrophage subtype. Some works associate the high expression of Fizz1 to pro-fibrogenic M2 macrophages^50^. Murthy *et al*.^51^ reported that macrophages with pro-fibrotic phenotype, positive for Arg-1, had reduced levels of Fizz1.

The present study also showed an important downregulation of hepatic CCR2 expression after monocyte transplantation. The CCR2 chemokine receptor is involved in migration of monocytes to lesion areas, and is highly expressed in Ly6C^hi^ monocytes/macrophages, stimulated during fibrosis progression^52^. A study conducted by Mitchell *et al*.^52^ demonstrated that mice with CCR2 knockout had significantly lower levels of CCl_4_-induced liver fibrosis compared to wild type animals. A reduction in hepatic expression of CCR2, eight weeks after a CD11b CD14^+^ monocyte infusion, may indicate a reduction of Ly6C^hi^ monocytes of pro-inflammatory profile in liver of the animals submitted to cell therapy, favoring the liver fibrosis improvement.

Recently, a new macrophage subtype has been identified, and it is considered a critical element for the resolution of liver fibrosis. Ramachandran and colleagues^22^ found in their investigations that Ly6C^low^ macrophages secrete large amounts of fibrolytic MMPs, as well as IL-10. Accordingly, an increase in MMP-9 and IL-10 levels were also observed in our study, suggesting that monocyte therapy influenced the regulation of activation pathways of macrophages involved in chronic inflammatory response. The markedly elevated hepatic IL-10 levels in animals treated with monocytes may alter the behavior of resident and recruited immune cells and the level of injury. Simultaneous up-regulation of IL-10 and MMP-9 following monocyte therapy may reduce HSCs activation^53^ and promote apoptosis^54^. Immune response modulation of liver injury through recruitment of host cells mediated by cytokines and chemokines, providing an anti-inflammatory microenvironment, has been proposed as an important mechanism of action associated with the beneficial effects of monocyte therapy.

Based our results, we can infer that monocyte therapy associated with PZQ chemotherapy, in a murine schistosomiasis model, seems to contribute to regression of liver fibrosis by a combination of mechanisms, which includes fibrous tissue ECM remodeling fibrosis by regulating MMPs secretion, suppression of pro-fibrogenic microenvironment in the liver and induction of inhibition of activated HSCs. Although there was no clear identification of a polarized macrophage activation profile, the findings observed suggest that the effects of the therapeutic modality proposed here are regulated by a predominance of M2reg macrophages. M2reg macrophages produce immunosuppressive cytokines (with a predominance of IL-10), and may be involved in the remodeling of fibrolytic proteins/ matrix, modulation of fibrogenesis and the inflammatory response resulting from hepatic tissue injury.

## Methods

### Animals

Sixty male C57BL/6 mice weighing 20–23 g were used in this study. Mice were purchased from the Centro de Criação de Animais de Laboratório (CECAL) Fundação Oswaldo Cruz (FIOCRUZ, Rio de Janeiro, Rio de Janeiro, Brazil), and housed in the animal research facility in the Instituto Aggeu Magalhães (IAM), (FIOCRUZ, Recife, Pernambuco, Brazil). They were acclimatized to a 12-hour light/dark cycle. The animals had free access to food and water. Animal care and the experiments were conducted in accordance with the approval of the Comissão de Ética no Uso de Animais (CEUA-IAM 15/2011).

### Experimental model of chronic schistosomiasis

Mice were submitted to infection by *S*. *mansoni*, transcutaneously, with 40 cercariae of LE strain (Belo Horizonte, Minas Gerais, Brazil). After 45 days, parasitological examination of feces was performed to confirm the infection. A period of 16 weeks was necessary for the development of the chronic phase of schistosomiasis.

### Chemotherapy

In the 16^th^ week post-infection, before the cell therapy, infected animals were subjected to the chemotherapeutic treatment with Praziquantel (PZQ), for elimination of adult worms. PZQ administration was given in a single oral dose at a concentration of 400 mg/kg of body weight.

### Isolation of bone marrow-derived monocytes

The bone marrows of femurs and tibia from C57BL/6 donor mice were extracted and used to obtain bone marrow mononuclear cells (BMMCs) by Ficoll gradient centrifugation (Histopaque 1119 and 1077, Sigma Aldrich, St. Louis, MO, USA). BMMCs were incubated with anti-CD11b antibodies conjugated to magnetic beads for monocyte isolation through an immunomagnetic separation system and phenotypic characterization as previously described^20^, resulting in a homogeneous CD11b CD14^+^ monocytes population.

### Experimental design

**Figure 8:**
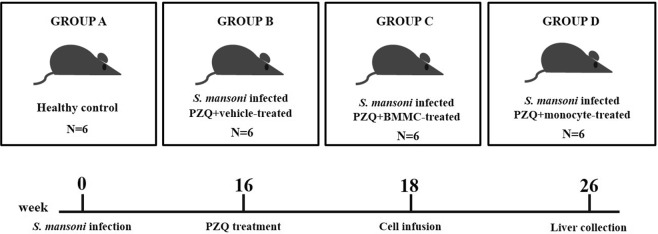
Structure of the experiment. Male C57BL/6 mice were submitted to subcutaneously infection by *S*. *mansoni* (40 cercariae). After 16 weeks of infection, the animals were submitted to the conventional chemotherapy with praziquantel (400 mg/Kg) (**B**, **C** e **D** groups). Cell therapy was initiated after 18 weeks of infection with 10^6^ bone marrow mononuclear cells (**C**) or 10^6^ monocytes (**D**). Group **B** received only saline (vehicle). Healthy uninfected mice were used as control (Group **A**). The therapeutic procedure was performed once a week for three consecutive weeks. Eight weeks after cell therapy (26^th^ week), the animals were submitted to euthanasia for liver collection (6 mice/group). The study data are a mean calculated from two independent experiments.

After the establishment of chronic schistosomiasis model, mice were randomly distributed into the following groups, with six mice in each group: *Group A:* Healthy control; *Group B*: Infected and vehicle-treated; *Group C:* Infected and BMMC-treated; *Group D:* Infected and Monocyte-treated. All groups with exception of the group A were treated with PZQ prior to vehicle or cell therapy. The study data are a mean calculated from two independent experiments. The experimental design is shown in Fig. 8.

### Cell infusion

Each mouse received three doses of 10^6^ BMMCs or monocytes suspended in 0.2 ml sterile saline (vehicle), intravenously. The procedure was performed once a week for three consecutive weeks. Eight weeks post-therapy, animals were submitted to euthanasia for liver collection.

### Morphometry

Specimens of the liver tissues were fixed in 10% formalin and embedded in paraffin wax. Sections (5-μm thick) were stained by Sirius-red and Fast green for collagen and granuloma analysis. Images of histological sections stained in Sirius Red were obtained by light microscope (DM LB 2, Leica Microsystems, Cambridge, UK) equipped with a LEICA JVC TK digital camera (model C 1380, Pine Brook, NJ, USA) and analyzed using the Image Analysis Processing System LEICA QWIN, version 2.6 MC (Leica Microsystems, Cambridge, UK). Ten microscopic fields (100X magnification) containing fibrous tissue areas were selected for quantification. From the histological sections stained with Sirius-red, some parameters of hepatic granuloma evaluation were also calculated: mean of numerical density (number of granulomas/unit volume) and mean volume of granuloma (mm^3^). Hepatic granulomas, well delimited and spherical shape which had the *S*. *mansoni* egg inside were considered during the measurement.

### Hydroxyproline mensuration

Liver samples (100–200 mg) were used to determine the hydroxyproline content (nMol/g tissue). Samples were processed and analyzed according to methodology previously described^55^, read at 558 nm in automatic spectrophotometer (Pharmacia Biotech, Sweden).

### Immunological assays

Cytokines and growth factors were quantified from the soluble liver protein extracts obtained as previously described^20^. Supernatants were used to quantify TNF-α, IL-6, IL-1β (BD OptEIA set mouse, San Diego, CA, USA), IL-13, IL-17, IL-23, MMP-9, TIMP-1 (R & D Systems, Minneapolis, MN, USA), TGF-β1, IL-10 (e-Bioscience, San Diego, CA, USA) and IL-4 (Invitrogen, California, USA) levels. Samples were read at a 450 nm wavelength using a microplate reader (model 3550, Thermo Scientific, Massachussetts, USA).

### Nitric Oxide (NO)

Soluble proteins extracts also were used for evaluation of NO levels by nitrites measurement using the Griess Kit (G7921, Molecular Probes, Oregon, USA), following the manufacturer’s instructions. Nitrite concentration was calculated in μM.

### Gene expression evaluation

Total RNA was extracted and purified from liver tissue using TRIzol™ Reagent (Invitrogen, California, USA). Complementary DNA was synthesized by reverse transcription (RT) with oligodT, using GoScript Transcription System (Promega, Wisconsin, USA). Sequences of primers are listed in Supplementary Table S1. Expression of mRNAs was assessed by qPCR using SYBR® Green PCR Master Mix (Applied Biosystems CA, USA) and ABI PRISM 7500 sequence detector (Applied Biosystems, CA, USA). qPCR was performed for the following targets: CCl5, IL-12β, CCR2 / Ly6C, Arginase 1, chitinase-3-like protein 3 (YM-1) Mannose 1 receptor-type C (CD206), Fizz1 (alpha 1 molecule Similar to resistin), α-SMA, TGF-β1 and Gal-3. Quantification was performed using the comparative ΔΔCT method, normalized with the reference gene, β-actin.

### Statistical analysis

Data were expressed as mean values ± standard error (SE). Initially, the quantitative data to the normality Test (Shapiro-Wilk) were submitted, indicating the Kruskal-Wallis non-parametric test, with Dunn *post hoc*. Statistical analyzes were performed using Graphpad Prism (version 5.0, San Diego, CA, USA). The P value < 0.05 was considered statistically significant.

## Supplementary information

Supplementary information

## Data Availability

The authors declare the availability of data.

## References

[1] Kumar A, Oati NT, Sarin SK (2011). Use Of Stem Cells For Liver Disease – Current Scenario. J. Clin. Exp. Hepatol..

[2] Novo E (2014). Cellular And Molecular Mechanisms In Liver Fibrogenesis. Arch Biochem Biophys..

[3] Zhang YC (2016). Liver Fibrosis And Hepatic Stellate Cells: Etiology, Pathological Hallmarks And Therapeutic Targets. World J. Gastroenterol..

[4] Schuppan D, Pinzani M (2012). Anti-Fibrotic Therapy: Lost In Translation?. J. Hepatol..

[5] Ali G, Masoud MS (2012). Bone Marrow Cells Ameliorate Liver Fibrosis And Express Albumin After Transplantation In CCl_4_-Induced Fibrotic Liver. Saudi. J. Gastroenterol..

[6] De Freitas Souza BS (2012). Transplantation Of Bone Marrow Cells Decreases Tumor Necrosis Factor-Α Production And Blood-Brain Barrier Permeability And Improves Survival In A Mouse Model Of Acetaminophen-Induced Acute Liver Disease. Cytotherapy.

[7] Salama H (2010). Autologous Hematopoietic Stem Cell Transplantation In 48 Patients With End-Stage Chronic Liver Diseases. Cell. Transplant..

[8] Lambertucci, J. R., Silva, L. C. S. & Voieta, I. Esquistossomose Mansônica. In *Dinâmica das doenças infecciosas e parasitárias* (Ed. Coura, J. R.) 931–946 (Guanabara Koogan; 2005).

[9] Oliveira SA (2008). Therapy With Bone Marrow Cells Reduces Liver Alterations In Mice Chronically Infected By *Schistosoma mansoni* Infection. World J. Gastroenterol..

[10] Elkhafif N (2008). Differentiation And Homing Of Transplanted Bone Marrow Cells In Livers Of Murine Schistosomiasis: Pilot Study. Aust. J. Basic and appl. Sci..

[11] El-Shennawy SF (2015). Therapeutic Potential of Mesenchymal Stem Cells on Early and Late Experimental Hepatic Schistosomiasis Model. J. Parasitol..

[12] Hegab MA (2018). Therapeutic Potential Effect Of Bone Marrow-Derived Mesenchymal Stem Cells On Chronic Liver Disease In Murine *Schistosomiasis mansoni*. J. Parasit. Dis..

[13] Hammam OA (2016). Wharton’s Jelly-Derived Mesenchymal Stem Cells Combined With Praziquantel As A Potential Therapy For *Schistosoma mansoni* Induced Liver Fibrosis. Sci. Rep..

[14] Lichtnekert J (2013). Changes In Macrophage Phenotype As The Immune Response Evolves. Curr. Opin. Pharmacol..

[15] Mori Y (2009). Participation Of Functionally Different Macrophage Populations And Monocyte Chemoacttractant Protein-1 In Early Stages Of Thioacetamide-Induced Rat Hepatic Injury. Toxicol. Pathol..

[16] Ju C, Tacke F (2016). Hepatic Macrophages In Homeostasis And Liver Diseases: From Pathogenesis To Novel Therapeutic Strategies. Cell. Mol. Immunol..

[17] Ramachandran P, Iredale JP (2012). Macrophages: Central Regulators Of Hepatic Fibrogenesis And Fibrosis Resolution. J. Hepatol..

[18] Wynn TA, Barron L (2010). Macrophages: Master Regulators Of Inflammation And Fibrosis. Semin. Liver Dis..

[19] Pellicoro A (2012). Elastin Accumulation Is Regulated At The Level Of Degradation By Macrophage Metalloelastase (MMP-12) During Experimental Liver Fibrosis. Hepatology.

[20] Souza VCA (2017). Bone Marrow-Derived Monocyte Infusion Improves Hepatic Fibrosis By Decreasing Osteopontin, TGF-Β1, Interleukin-13 And Oxidative Stress. World J. Gastroenterol..

[21] Mahbub S, Deburghgraeve CR, Kovacs EJ (2012). Advanced Age Impairs Macrophage Polarization. J. Interferon Cytokine Res..

[22] Ramachandran P (2012). Differential Ly-6C Expression Identifies The Recruited Macrophage Phenotype, Wich Orchestrates The Regression Of Murine Liver Fibrosis. Proc. Natl. Acad. Sci. USA.

[23] Thomas JA (2011). Macrophage therapy for murine liver fibrosis recruits host effector cells improving fibrosis, regeneration, and function. Hepatology.

[24] Tanimoto H (2013). Improvement Of Liver Fibrosis By Infusion Of Cultured Cells Derived From Human Bone Marrow. Cell Tissue Res..

[25] Duffield JS (2013). Host Responses in Tissue Repair and Fibrosis. Annu. Rev. Pathol. Mech. Dis..

[26] Yao L (2015). Association Between Interleukin-10 Gene Promoter Polymorphisms And Susceptibility To Liver Cirrhosis. Int. J. Clin. Exp. Pathol..

[27] Hammerich L, Tacke F (2014). Interleukins In Chronic Liver Disease: Lessons Learned From Experimental Mouse Models. Clin. and Exp. Gastroenterol..

[28] Suh YG (2012). CD11b(+) Gr1(+) Bone Marrow Cells Ameliorate Liver Fibrosis By Producing Interleukin-10 In Mice. Hepatology.

[29] Rutitzky LL, Stadecker MJ (2011). Exarcebated Egg-Induced Immunopatology In Murine *Schistosoma Mansoni* Infection Is Primarily Mediated By IL-17 And Restrained By IFN-Gamma. Eur. J. Immunol..

[30] Wang L, Chen S, Xu K (2011). Il-17 Expression Is Correlated With Hepatitis B Related Liver Diseases And Fibrosis. Int. J. Mol. Med..

[31] Larkin (2012). Induction And Regulation Of Pathogenic Th17 Cell Responses In Schistosomiasis. Semin. Immunopathol..

[32] Braga, T. T., Agudelo, J. S. H. & Camara, N. O. S. Macrophages During The Fibrotic Process: M2 As Friend And Foe. *Front*. *Immunol*. **6**(602) (2015). 10.3389/fimmu.2015.00602 PMC4658431 26635814

[33] Ndlovu H (2018). Interleukin-4 Receptor Alpha Expressing B Cells Are Essential to Down-Modulate Host Granulomatous Inflammation During Schistosomasis. Front Immunol..

[34] Moghaddam A (2018). Macrophage plasticity, polarization, and function in health and disease. J Cell Physiol.

[35] Chuah C (2014). Cellular and chemokine-mediated regulation in schistosome-induced hepatic pathology. Trends Parasitol..

[36] Yang L (2014). Vascular Endothelial Growth Factor Promotes Resolution And Repair In Mice. Gastroenterology.

[37] Pham Van T (2008). Expression Of Matrix Metalloproteinase-2 And -9 And Of Tissue Inhibitor Of Matrix Metalloproteinase-1 In Liver Regeneration From Oval Cells In Rat. Matrix Biol..

[38] Li LC, Li. J, Gao J (2014). Functions Of Galectin-3 And Its Role In Fibrotic Diseases. J. Pharmacol. Exp. Ther..

[39] Breuilh l (2007). Galectin-3 Modulates Immune and Inflammatory Responses during Helminthic Infection: Impact of Galectin-3 Deficiency on the Functions of Dendritic Cells. Infect. Immun..

[40] Traber PG, Zomer E (2013). Therapy Of Experimental Nash And Fibrosis With Galectin Inhibitors. Plos One.

[41] Traber PG (2013). Regression Of Fibrosis And Reversal Of Cirrhosis In Rats By Galectin Inhibitors In Thioacetamide-induced Liver Disease. PLos One.

[42] Oliveira SA (2012). Reduction Of Galectin-3 Expression And Liver Fibrosis After Cell Therapy In A Mouse Model Of Cirrhosis. Cytotherapy.

[43] Tacke F, Zimmermann HW (2014). Macrophage Heterogeneity In Liver Injury And Fibrosis. J. Hepatol..

[44] Wynn TA, Ramalingam TR (2012). Mechanisms Of Fibrosis: Therapeutic Translation For Fibrotic Disease. Nat. Med..

[45] Lech M, Anders HJ (2013). Macrophages And Fibrosis: How Resident And Infiltrating Mononuclear Phagocytes Orchestrate All Phases Of Tissue Injury And Repair. Biochim. Biophys. Acta..

[46] Munder M (2009). Arginase: An Emerging Key Player In The Mammalian Immune System. Brit. J. Pharmacol..

[47] Wang Y (2015). Increases Of M2a Macrophages And Fibrosis In Aging Muscle Are Influenced By Bone Marrow Aging And Negatively Regulated By Muscle-Derived Nitric Oxide. Aging. Cell.

[48] Pesce JT (2006). The IL-21 Receptor Augments Th2 Effector Function And Alternative Macrophage Activation. J. Clin. Invest..

[49] Beljaars, L. *et al*. Hepatic Localization Of Macrophage Phenotypes During Fibrogenesis And Resolution Of Fibrosis In Mice And Humans. *Front*. *Immunol*. **5**(430) (2014). 10.3389/fimmu.2014.00430 PMC4157549 25250030

[50] Wilson MS (2007). Immunopathology Of Schistosomiasis. Immunol. Cell. Biol..

[51] Murthy S (2017). Alternative Activation Of Macrophages And Pulmonary Fibrosis Are Modulated By Scavenger Receptor, Macrophage Receptor With Collagenous Structure. The FASEB Journal.

[52] Mitchell C (2009). Dual Role of CCR2 in the Constitution and the Resolution of Liver Fibrosis in Mice. Am. J. Pathol.

[53] Lan L (2008). Transplantation of bone marrow-derived hepatocyte stem cells transduced with adenovirus-mediated IL-10 gene reverses liver fibrosis in rats. Transpl Int..

[54] Zhou X (2004). Engagement of alphavbeta3 integrin regulates proliferation and apoptosis of hepatic stellate cells. J. Biol. Chem..

[55] Bergman I, Loxley R (1963). Two Improved And Simplified Methods For The Spectrophometric Determination Of Hydroxyproline. Anal. Chem..

