# Supplementary material for: Adoptive Transfer of Bone Marrow-Derived Monocytes Ameliorates Schistosoma mansoni -Induced Liver Fibrosis in Mice
Source: Sci Rep. 2019 Apr 23;9:6434. doi: 10.1038/s41598-019-42703-y (PMC6478942; doi:10.1038/s41598-019-42703-y)
Supplement: Supplementary file 1 — Supplementary information [file 41598_2019_42703_MOESM1_ESM.pdf]

Adoptive Transfer of Bone Marrow-Derived Monocytes Ameliorates *Schistosoma mansoni* -Induced Liver Fibrosis in Mice.

Veruska Cintia Alexandrino de Souza[1], Danielle Maria Nascimento Moura[1], Maria Carolina Accioly Brelaz de Castro[2], Patrícia Torrez Bozza[3], Ligia de Almeida Paiva[3], Camila Juliet Barbosa Fernandes[1], Renata Lins Carneiro Leão[1], Jéssica Paula Lucena[1], Roni Evencio de Araújo[1], Alex José de Melo Silva[1], Regina Celia Bressan Queiroz Figueiredo[1], Sheilla Andrade de Oliveira[1*].

Supplementary information

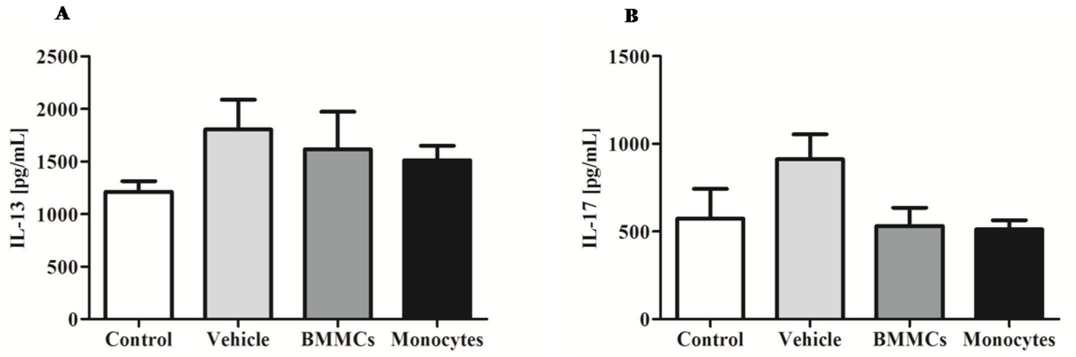

Supplementary Figure S1. Evaluation of effects of Monocyte therapy in murine model of schistosomiasis. (A) IL-13 and (B) Il-17 hepatic tissue levels, quantified by by sandwich ELISA. (n=6 mice/group).

Gene	Sequence
**CCL5**	Forward 5’-CCAGAGAAGAAGTGGGTTCAAG-3’
	Reverse 5’-AGCAATGACAGGGAAGCTATAC-3’
**IL-12β**	Forward 5’-GTAACCAGAAAGGTGCGTTCC-3’
	Reverse 5’-GAACACATGCCCACTTGCTG-3’
**Arg-1**	Forward 5’-CCAGGGACTGACTACCTTAAAC-3’
	Reverse 5’-GAAGGCGTTTGCTTAGTTCTG-3’
**YM-1**	Forward 5’-ACCATGGCCAAGCTCATT-3’
	Reverse 5’-GTCCTTAGCCCAACTGGTATAG-3’
**CD206**	Forward 5’-GTTCACCTGGAGTGATGGTTCTC-3’
	Reverse 5’-GACATGCCAGGGTCACCTTT-3’
**Fizz1**	Forward 5’-ACTTGCAACTGCCTGTGCTTAC-3’
	Reverse 5’-TCAAAGCTGGGTTCTCCACCTC-3’
**CCR2**	Forward 5’-CAAATCAAAGGAAATGGAAGACAAT-3’
	Reverse 5’-GCCCCTTCATCAAGCTCTTG-3’
**α-SMA**	Forward 5’-TCAGGGAGTAATGGTTGGAATG-3’
	Reverse 5’-GGTGATGATGCCGTGTTCTA-3’
**TGF-β1**	Forward 5’-GGTGGTATACTGAGACACCTTG-3’
	Reverse 5’-CCCAAGGAAAGGTAGGTGATAG-3’
**Gal-3**	Forward 5’-CTGAGAGATACCCATCGCTTTG-3’
	Reverse 5’-GTAGCTCAGTGAGAGAACACTT-3’
**B-actina**	Forward 5’-CCGTAAAGACCTCTATGCCAAC-3’
	Reverse 5’-AGGAGCCAGAGCAGTAATC-3’

Supplementary Table S1. Sequence of murine primers used for quantitative real time polymerase chain reaction (qPCR). Abbreviations: Arg, arginase; Gal, galectin.
